# Immunometabolic Regulation of Interleukin-17-Producing T Helper Cells: Uncoupling New Targets for Autoimmunity

**DOI:** 10.3389/fimmu.2017.00311

**Published:** 2017-03-21

**Authors:** Katrina J. Binger, Beatriz F. Côrte-Real, Markus Kleinewietfeld

**Affiliations:** ^1^Department of Biochemistry and Molecular Biology, Bio21 Institute of Molecular Science and Biotechnology, University of Melbourne, Parkville, VIC, Australia; ^2^VIB Laboratory of Translational Immunomodulation, Hasselt University, BIOMED, Diepenbeek, Belgium

**Keywords:** glycolysis, oxidative phosphorylation, immunometabolism, interleukin-17-producing T helper cells, regulatory T cells, autoimmune diseases

## Abstract

Interleukin-17-producing T helper (Th17) cells are critical for the host defense of bacterial and fungal pathogens and also play a major role in driving pathogenic autoimmune responses. Recent studies have indicated that the generation of Th17 cells from naïve CD4^+^ T cells is coupled with massive cellular metabolic adaptations, necessary to cope with different energy and metabolite requirements associated with switching from a resting to proliferative state. Furthermore, Th17 cells have to secure these metabolic adaptations when facing nutrient-limiting environments, such as at the sites of inflammation. Accumulating data indicates that this metabolic reprogramming is significantly linked to the differentiation of T helper cells and, particularly, that the metabolic changes of Th17 cells and anti-inflammatory Forkhead box P3^+^ regulatory T cells are tightly and reciprocally regulated. Thus, a better understanding of these processes could offer potential new targets for therapeutic interventions for autoimmune diseases. In this mini-review, we will highlight some of the recent advances and discoveries in the field, with a particular focus on metabolic demands of Th17 cells and their implications for autoimmunity.

## Introduction

T cells respond to alterations in the host environment *via* multiple steps of activation, proliferation, and finally differentiation into specialized subsets, which are exquisitely programmed to deal with the challenge at hand; whether it is the presence of an intracellular or extracellular pathogen, or other alterations in tissue homeostasis, which may require either inflammatory or regulatory responses. These various responses are tightly regulated by the interplay of specialized CD4^+^ effector T helper (Th) cell subsets and antiinflammatory Forkhead box P3^+^ (FOXP3^+^) regulatory T cells (Tregs) (1).

One such type of a specialized T effector cell subset consists of interleukin (IL)-17-producing T helper (Th17) cells. In mice, Th17 cells could be differentiated from stimulated naive CD4^+^ T cells in the presence of the pleiotropic cytokine transforming growth factor (TGF)-β1 in combination with IL-6. Th17 cells are specialized to respond against certain bacterial and fungal pathogens in the tissue sites in which they are located, namely the mucosal linings of the gut and airway epithelia (2). However, Th17 cells are also known for their pathogenic potential against the host, due to their association with several autoimmune diseases such as multiple sclerosis (MS), psoriasis, and rheumatoid arthritis (RA) (1–4). More recent studies have demonstrated that Th17 cells can be heterogeneous in phenotype and function and can even show antiinflammatory properties. This pro-inflammatory versus antiinflammatory/homeostatic phenotype of Th17 cells seems to be determined by a set of specific signaling modules, where pathogenicity is critically influenced by a high level of expression of IL-23 receptor, granulocyte-macrophage colony-stimulating factor, and Th1-like transcripts [e.g., interferon-γ, T-box transcription factor 21 (TBX21/Tbet)] and by the absence of the antiinflammatory cytokine IL-10 (5). The induction of these different Th17 phenotypes can be mimicked *in vitro* by varying the combination of stimulatory triggers and cytokines. For example, the stimulation of naive T cells with a combination of IL-1β, IL-6, and IL-23 in the absence of TGF-β1 induces the differentiation of Th17 cells that exhibit a highly pro-inflammatory and pathogenic phenotype, compared to “classically” TGF-β1 + IL-6 differentiated Th17 cells (6–8). Together, these studies illustrate that the pro-inflammatory potential of Th17 cells is extremely sensitive to the presence and combinations of stimulatory cues within the local microenvironment.

Of course *in vivo*, the tissue site in which Th17 cells are generated does not only consist of cytokines but is also packed with a variety of small molecules, including metabolites. This “metabolomic” *milieu* is dynamically changing in both its composition and concentration, which may have various effects on Th17 cells, particularly through their cellular metabolism (9–12). This is highlighted by many recent studies showing even further variation in Th17 phenotype can been fashioned *in vitro* by the addition of e.g., fatty acids (13, 14), phospholipids (15), cholesterol intermediates (16), oxysterols (17, 18), and even electrolytes such as sodium or potassium (19, 20). In this mini-review, we will highlight some recent advances in understanding the metabolic adaptations and mechanisms employed by T cells when they undergo activation and differentiate into specialized subsets, focusing on Th17 cells.

## Metabolic Adaptations of Th Cells

The overall aim of cellular metabolism, independently of the cell type, is to generate energy [adenosine triphosphate (ATP)] and metabolites, which are essential for cells to perform various functions, sustain life and growth. Glucose is the major cellular fuel source, and it is broken down into ATP by two separate, but connected pathways: glycolysis and oxidative phosphorylation (OXPHOS). In glycolysis, glucose is broken *via* 10 enzymatic steps down to pyruvate, yielding two ATP molecules; a process that does not require oxygen. Most cells go on to oxidize pyruvate in the tricarboxylic acid cycle, subsequently fueling mitochondrial OXPHOS, which, in an oxygen-dependent process, yields more than 30 ATP. Alternatively, pyruvate can be converted to lactate, which ultimately feeds back into glycolysis. While glycolysis produces far less ATP, it has several advantages in that it is fast and generates metabolites, so under oxygen-poor conditions, it can suffice to provide energy and necessary biomolecules for the cell (11, 12, 21).

**Figure 1:**
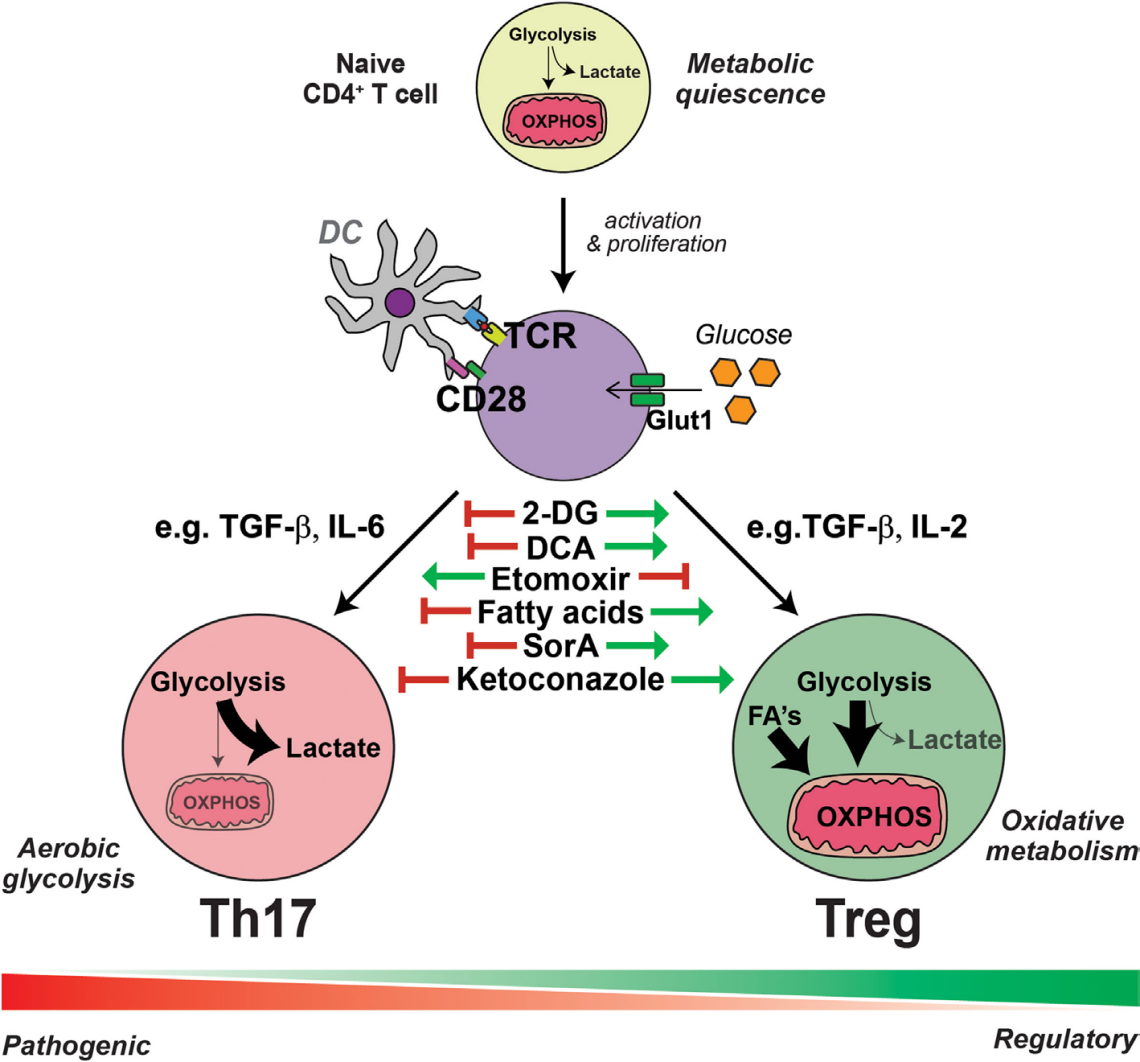
**Interleukin-17-producing T helper (Th17) cells and regulatory T cells (Tregs) have distinct metabolic requirements**. The presentation of antigen and costimulation by dendritic cells induce the reprogramming of T cells into an aerobic glycolytic “Warburg” metabolism, characterized by the increased expression of glucose transporter 1 (Glut1) and augmented glucose uptake. Generally, in the presence of transforming growth factor (TGF)-β with the combination of other cytokines such as interleukin (IL)-6, activated T cells are polarized into specialized Th17 cells, which have an even further enhanced glycolytic metabolism and flux into intermediate pathways, required for the generation of amino acids, nucleotides, and fatty acids essential for Th17 effector function. Conversely, the induction of Tregs from activated T cells by TGF-β and the combination of other cytokines, such as IL-2, results in an increased uptake of fatty acids (FA) and an elevated lipid and mitochondrial oxidative metabolism. Within each subset, there exists further heterogeneity, depending on the presence of further local stimulatory cues, such as other cytokines and small metabolites, thus generating pathogenic or non-pathogenic Th17 cells and various polarized regulatory Treg cells; all of which exhibit further metabolic complexity. Th17 cells are dependent on aerobic glycolytic metabolism, as inhibiting glycolysis with 2-deoxyglucose (2-DG) or dichloroacetate (DCA) prevents Th17 cell generation even in the presence of Th17 cell-promoting conditions. Likewise, the addition of exogenous fatty acids or soraphen A (SorA), which enhances lipid oxidative metabolism, similarly inhibits Th17 cell generation. These metabolic interventions have the opposite effect on the generation of Tregs, which are conversely enhanced by treatments that augment lipid oxidative metabolism and blunted by inhibitors of lipid transport such as etomoxir. Cholesterol derivatives are essential for Th17 cell differentiation and blockade of cholesterol biosynthesis, for example, with ketoconazole, inhibits the generation of Th17 cells but has no effect on Tregs.

A metabolic phenomena first observed in cancerous cells by Otto Warburg in 1924 is when proliferating cells preferentially utilize glycolytic metabolism even when oxygen is abundant (22). This aerobic glycolysis, often called “Warburg” metabolism, while bioenergetically less efficient, results in an additional benefit from increased flux into new molecules (nucleotides, amino acids, and fatty acids), as well as pathways involved in generation of redox-protective metabolites. It is now apparent that the activation of T cells induces this same metabolic switch to aerobic glycolysis (Figure 1). In fact, this was first observed more than 50 years ago that the activation of T cells, such as with mitogens like concanavalin A, results in immense changes in glycolytic cellular metabolism (23–27). More recently, Frauwirth et al. demonstrated the importance of costimulation in initiating these metabolic adaptations (28). Costimulation of CD3 and CD28 induced increased expression of the glucose transporter 1 (Glut1), subsequently leading to augmented glucose uptake and flux through glycolysis. The same has been reported for the expression of amino acid transporters and amino acid uptake (29, 30). Functionally, this provides the activated T cell with ATP, but more importantly, the accumulation of nutrients and biomass permits the activated T cell to expand and rapidly proliferate. Adopting an aerobic glycolytic metabolism is now appreciated to be a feature of all Th cell populations (9–11, 21). Furthermore, this switch to aerobic glycolysis is not only limited to proliferating immune cells, as the activation of myeloid cells like dendritic cells (31) and macrophages (32) with toll-like receptor agonists also induces these same metabolic adaptations, indicating that this mechanism may infer a general requirement for cells of the immune system.

## Th17 Cells Versus Treg: A Choice in Cellular Metabolism

For differentiation into special T effector cell subsets, cellular metabolism remains a process that is distinct and necessary for the acquisition of downstream specialized functions. This is none the more apparent when comparing the metabolic configurations of Th17 cells and Tregs. As a consequence of costimulation in combination with TGF-β and IL-6, the transcription factor RAR-related orphan receptor gamma t (RORγt) is activated leading to the generation of Th17 cells. Conversely, while maintaining a requirement for TGF-β in the absence of other cytokines, with the exception of IL-2, induced Tregs (iTregs) are generated *via* the activation of the transcription factor FOXP3. Metabolically, these two subsets are incredibly distinct (13, 33, 34). *In vitro* stimulation of naïve T cells with Th17-stimulating conditions induces robust glucose uptake and a shift to aerobic glycolysis. By contrast, Tregs instead increase their lipid uptake and utilize energy-efficient pathways such as fatty acid oxidation (FAO) and mitochondrial OXPHOS (Figure 1) (10–12, 21).

The key factors essential for the regulation of Treg and Th17 cell metabolic reprogramming have now been identified as a network of metabolic kinases that function as nutrient/energy sensors and metabolic transcription factors, in line with their regulation of nutrient transporter expression (9–12, 21, 35). Major regulatory checkpoints are mechanistic/mammalian target of rapamycin (mTOR), the kinase complex that is a promoter of aerobic glycolysis and anabolic metabolism after stimulation of T cells and the AMP-activated protein kinase (AMPK) complex that promotes FAO and catabolic metabolism [reviewed in Ref. (9–12, 21, 35)]. Particularly for Th17 cells, the metabolic transcription factor, hypoxia-inducible factor 1 (HIF-1α), seems to play a special role (36). Under Th17-promoting conditions, HIF-1α expression is rapidly increased in an mTOR-dependent manner, and its deletion prevents the generation of Th17 cells both *in vitro* and in Th17-promoting disease models *in vivo* (34, 37). As HIF-1α is a transcription factor regulating the expression of metabolic enzymes (36), Shi et al. hypothesized its importance in regulating the cellular metabolic reprogramming of Th17 cells (34). Deletion of HIF-1α under Th17-promoting conditions results in a blunted upregulation of Glut1 and the reduced expression of crucial glycolytic enzymes such as hexokinase 2, phosphofructokinase 1, and lactate dehydrogenase. Together, these data indicate that indeed HIF-1α is an essential facilitator of the acquisition of Th17 glycolytic metabolism (34). In line with this, a recent study identified another important regulatory checkpoint for Th17 cell or Treg-specific metabolic pathway decisions. Gerriets et al. identified pyruvate dehydrogenase (PDH) and the pyruvate metabolism as a key decisive point between T cell glycolytic and oxidative metabolism. The conversion of cytosolic pyruvate into mitochondrial acetyl-CoA is catalyzed by PDH for oxidative metabolism and is inhibited by PDH kinase (PDHK). PDHK is regulated by hypoxia and HIF-1α and promotes the generation of lactate by suppressing pyruvate oxidation (38). By a detailed metabolic analysis, they identified with PDHK1 an isoform that is predominantly expressed in Th17 cells but not in Th1 cells or Tregs. The inhibition of PDHK1 by dichloroacetate (DCA) was able to suppress glycolysis and selectively affected the generation and survival of Th17 cells in part through the generation of reactive oxygen species (ROS) (38).

By contrast, both *in vitro* generated iTregs and *ex vivo* isolated thymic-derived Tregs exhibit increased phosphorylation of AMPK (13), a broad sensor of decreased cellular nutrients and energy (39). Subsequently, treatment *in vivo* with an AMPK agonist, metformin, resulted in an increased generation of Tregs (13). However, conversely, AMPK-knockout CD4^+^ T cells did not demonstrate deficient Treg generation (40). An explanation for these discrepant results may be because AMPK has a much broader role in T cell metabolic adaptations, not only within the Treg subset. Recently, Blagih et al. showed that T cells deficient in AMPK are unable to adapt *in vitro* changes in the availability of nutrients such as glucose and glutamine. Subsequently, this translated to an impaired ability of both Th1 and Th17 generation *in vivo* (41), pointing toward multiple layers of cellular metabolic sensing and adaptation in the acquisition of T effector function, particularly for Th17 cells. In line with this, another metabolic checkpoint for fate decisions between Tregs and Th17 cells was recently described (33). *De novo* fatty acid synthesis (FAS), which is inhibited by AMPK (42), was shown to be essential for the generation of Th17 cells in contrast to Tregs (33). Accordingly, the inhibition or deletion of acetyl-CoA carboxylase 1 (ACC1), a key enzyme for *de novo* FAS, resulted in an impaired Th17 differentiation, whereas Tregs were induced (21, 33, 43).

These findings highlight how the generation of Tregs and Th17 cells is tightly linked to their metabolic state, offering potential new targets for the regulation of these two reciprocally regulated T cell subsets (Figure 1). Indeed, it was shown already in addition to the targeting of AMPK with metformin that the inhibition of mTOR by rapamycin could block Th17 cell generation while favoring Tregs (11). Moreover, the inhibition of lipid oxidation with drugs such as etomoxir, which inhibits the activity of carnitine palmitoyltransferase I, resulting in a reduction of the fatty acid import into the mitochondria (44), impairs the differentiation of iTregs but has no effect on the generation of Th17 cells (13, 44). Conversely, inhibiting glucose metabolism with 2-deoxyglucose impairs Th17 cell differentiation while reciprocally promoting iTreg induction (34). Similarly, the addition of exogenous fatty acids (13) or the inhibition of *de novo* FAS with small-molecule metabolic modulators like soraphen A (SorA) (33) impairs Th17 cell differentiation and promotes Tregs, even in the presence of pro-Th17-inducing conditions. This does not mean, however, that Th17 cells do not require lipids for their metabolic remodeling. It has been recently shown that oxysterols, such as 27-dihydroxycholesterol (18), metabolites in the cholesterol biosynthetic pathway (16), and desmosterol (17) bind to, and agonize RORγt activity, subsequently promoting the differentiation of Th17 cells. Thus, inhibiting cholesterol synthesis, for example with ketoconazole, impairs Th17 differentiation and IL-17 production, but has no effect on Treg differentiation (17). Moreover, PDHK1 blockade by DCA was able to selectively impair Th17 cell function and ameliorated experimental autoimmune encephalomyelitis (EAE) without having significant effects on Tregs or Th1 cells (38).

## Th17 Metabolism: A Potential Therapeutic Target for the Treatment of Autoimmune Diseases?

Under homeostatic conditions, Th17 cells are easily able to undergo the critical metabolic changes required for their specialized function described above, as nutrients are plentiful. However, under altered conditions (e.g., infection, disease, diet), metabolic restrictions may occur, which could have significant bearings on Th17 cell function. These recent discoveries discussed before highlight the intimate connectivity between immunity and cellular metabolism leading to the following questions: (a) Do disturbances in T cell metabolism contribute to the development of human autoimmune diseases? (b) Can Th17 metabolism be therapeutically targeted, thereby manipulating the immune system to become less inflammatory, and thus providing protection or treatment for autoimmune diseases with strong Th17 components such as RA and MS?

Besides in animal models of disease, it has additionally been demonstrated that several autoimmune diseases go in line with metabolic alterations and that also T cells of patients have dysregulated metabolic profiles, indicating that indeed an altered T cell metabolism is associated with disease. This was for instance already shown for RA, systemic lupus erythematosus, and MS (11, 12, 45–47). Albeit for most of the diseases premature and incompletely understood, the notion of altered metabolic profiles in human autoimmunity points towards intriguing new avenues for therapeutic interventions. In fact, recent studies have generated promising experimental data indicating the feasibility of this hypothesis. Most of the current reports concentrated here on the reciprocal regulation in regards of cellular metabolism between Tregs and Th17 cells, since Th17 cells primarily depend on glycolysis and Tregs seem to utilize mainly FAO. As described above, the key targets here would be the inhibition of the mTOR/HIF-1α pathway to prevent the development of the initiation of a “pro-inflammatory” metabolic signature or the activation of AMPK to influence cellular metabolism favoring the generation of antiinflammatory Tregs. In line with this, the interference of *de novo* FAS by inhibiting ACC1 with the inhibitor SorA was demonstrated to be effective at promoting the generation of Tregs, as well as inhibiting Th17 cells (33). However, the major problem, as for many other currently available immunotherapies, remains specificity. The above-mentioned metabolic reactions are all important in other T cell subsets and immune cells, and therefore, unspecific targeting of these pathways bears the risk of general immunosuppression and/or the interference with the generation of memory cells, which seem to have similar metabolic demands as Tregs; not to mention effects on non-immune cells (9, 10, 12, 21). Moreover, it remains to be seen on how other Th cell subsets react and in addition, importantly, on how these pathways interfere with pathogenic and regulatory/homeostatic subsets of Th17 cells (5). In this respect, it is of interest that it has already been demonstrated that an altered lipid biosynthesis and the fatty acid and cholesterol composition of the cell could impact Th17 cell pathogenicity by generating endogenous RORγt ligands (15–18).

It is also important to note that for many of the studies described in this review, there are only sparse data on the contribution of these same metabolic pathways to the differentiation of human Tregs and Th17 cells, and the contribution of these molecules to disease *in vivo* is not clear. Recent studies have, for instance, shown that in contrast to findings in murine Tregs, human Tregs in fact highly depend on glycolysis (46, 48). Similarly, murine Tregs have also been shown to be glycolytic *in vivo* (49). This obvious discrepancy of human to murine Tregs might be associated with the existence of different *FOXP3* splice variants in humans, which could dictate their function in contrast to mice (50, 51). It was shown that glycolysis through the glycolytic enzyme enolase-1 controls the induction of the *FOXP3* splice variant containing exon 2 that is crucial for human Treg function (46). Therefore, defining metabolic targets depending only on *in vitro* findings and experimental animal models must be carefully examined if they can be fully translated to the setting of human disease. Promising experimental strategies to cope with these problems might be the inclusion of different mouse models expressing different *FOXP3* splice variants or the use of humanized mouse models, in combination with the analysis of patient samples as recently demonstrated by Yang et al. for RA (52). This elegant study identified that patient samples exhibited a dysregulation in the pentose phosphate pathway (PPP), which was associated with a depletion in ROS. Insufficient oxidative signaling resulted in ataxia telangiectasia mutated kinase-dependent bypass of the G_2_/M cell cycle, consequently leading to hyperproliferation and a shift of RA patients into a highly pathogenic Th17/Th1 cell profile. Importantly, this dysregulation could be rescued by metabolic interventions that restored the ROS pool: supplementation with menadione (vitamin K3), disrupting synthesis of the ROS quencher glutathione, or by blocking glucose shunting into the PPP, which was even demonstrated in a humanized mouse model *in vivo*, transplanted with synovial tissue and T cells of RA patients (47, 52).

## Conclusion

The research on immunometabolism and its relation to disease is an extremely fast growing and promising field, which may offer novel therapeutic avenues for immunomodulation in settings of various human diseases. Particularly in autoimmune diseases like RA or MS, metabolic disturbances influencing the Treg/Th17 axis may play a role, and we are just at the beginning to understand the tight linkage between metabolic pathways and immune cell function.

While this increased knowledge of the metabolic requirements and alterations of Th17 cells and Tregs in autoimmunity provides many specific targets for intervention, it may also offer indirect dietary approaches that beneficially influence dysregulated T cell metabolism by systemically changing nutrient or metabolite availability. This is not a new concept, as it is well known that nutrition and the general metabolic condition of individuals has a profound impact on the immune system. Malnutrition, particularly prominent in developing countries, is clearly associated with reduced immune function. By contrast, obesity, which is associated with a “Western” lifestyle and a nutritional pattern high in calories, fat, and salt, goes in hand with the onset of a low-grade systemic inflammation (12, 53, 54). While obesity is well known to predispose individuals to diabetes and cardiovascular diseases, it additionally seems to represent a risk factor for inflammatory autoimmune diseases like MS (53, 55, 56). This may be driven largely by the strong effect of obesity on the Treg/Th17 axis through various metabolites or adipokines like leptin and through pro-inflammatory cytokines like IL-6 (53, 57). An additional molecular mechanism contributing to this effect was recently described by Endo et al. Here, mice fed a high-fat diet had an elevated induction of Th17 differentiation, in a mechanism that was reliant on the expression of ACC1, which subsequently contributed to RORγt activation (43). Therefore, it is important to understand the direct and indirect influences of diet on the immune system and Treg/Th17 axis, or more broadly, the connection of systemic and cellular metabolism and its relation to human autoimmune disease (45). A variety of studies have indicated that particularly dietary interventions could have severe effects on autoimmunity by influencing metabolic parameters of immune cells. It is thus tempting to speculate that, for instance, dietary interventions like fasting mimicking diets, that were recently shown to be beneficial in EAE and MS patients by shifting the Treg/Th17 balance (58), or by lowering the sodium content in diets, that had a strong metabolic impact on macrophages (59, 60), could serve as interesting alternatives or additions for drugs directly targeting cellular metabolism.

## Author Contributions

All authors listed have made substantial, direct, and intellectual contribution to the work and approved it for publication.

## Conflict of Interest Statement

The authors declare that the research was conducted in the absence of any commercial or financial relationships that could be construed as a potential conflict of interest.
